# Remote Relaxation and Acceptance Training for the Management of Stress in Cancer Patients: A Study Protocol

**DOI:** 10.3389/fpsyg.2021.710861

**Published:** 2021-10-13

**Authors:** Chiara Marzorati, Silvia Francesca Maria Pizzoli, Roberto Grasso, Gabriella Pravettoni

**Affiliations:** ^1^Applied Research Division for Cognitive and Psychological Science, European Institute of Oncology IRCCS, Milan, Italy; ^2^Department of Oncology and Hemato-Oncology, University of Milan, Milan, Italy

**Keywords:** remote training, social isolation, stress management, cancer, relaxation, acceptance therapy

## Abstract

**Background:** Cancer patients are now facing a double distinctive challenge of survival against both the disease and fear of contracting COVID-19. This challenge has resulted in the forced adoption of social distancing measures and reorganization of the delivery of medical and psychological treatments. The perceived loneliness and uncertainty increased distress and symptoms burden. In the current period, eHealth interventions might provide valuable benefits in the field of cancer care.

**Objective:** The overall goal of the study protocol will be to provide an innovative intervention for cancer patients based on an online platform, to help them manage and prevent psychological problems related to social isolation. Specifically, the efficacy of two web-based interventions aimed at lowering stress in cancer patients will be tested and compared.

**Methods:** One hundred and fifty participants (75 per group) will be enrolled in a two-group randomized trial. The two interventions will be composed either by exercises on relaxation and meditation practices, presented in both automated online content and interactive group sessions or by fixed psychoeducational online content. Stress, anxiety, and depressive symptoms, distress, resilience, and perceived social isolation will be measured before the start of the interventions (T0), 2 weeks (T1), 4 weeks (T2), and 2 months (T3) after the beginning of the interventions in both groups. A repeated measures ANOVA will be performed to test differences in the questionnaires' scores between groups across the four-time points.

**Expected Results:** We hypothesized greater improvement in the specific domain of stress symptoms (IES-R) assessed in the group receiving the interactive intervention, compared to the group which will receive only fully automated psychoeducational content. Secondarily, we expect the same trend of improvement across all the psychological variables in the blended intervention group.

**Conclusions:** Implementing these practices on people who are forced into mandatory social isolation may help them become more aware of their mind-body condition and reduce negative effects. Moreover, relaxation techniques help individuals in achieving a greater state of well-being, increasing the ability to cope with stressful situations (resilience), and strengthening the immune system.

## Introduction

The COVID-19 pandemic has spread worldwide. It has been 1 year since Italy has forced a strict lockdown, with a drastic reorganization of the healthcare system. To contain the extensive viral spread, hospitals are implementing several measures to cope with the demand for care and adequately address patients' needs (van de Haar et al., 2020). The risk of exceeding healthcare systems' capacity has led to the adoption of social distancing measures and the reorganization of medical treatment delivery. Further restrictions were adopted in hospitals such as limiting elective surgical interventions, restricting caregivers' visits during hospital stays, reducing systemic treatment intensity, and improving teleconsultations (Sigorski et al., 2020). This is even more difficult with people with a higher risk of getting infected. In particular, the scientific studies showed that patients with cancer were more vulnerable to both the SARS-CoV-2 infection and its complications (Emami et al., 2020).

Cancer patients are facing a double distinctive challenge of survival against both the disease and fear of contracting the virus. The perception of physical vulnerability to the illness enhanced arousal and triggered the anxiety. Patients are overwhelmed by conflicting advice and fear of cancer recurrence (Miaskowski et al., 2020). The lack of social connections may enhance patients' feelings of loneliness and uncertainty and they may contribute to the exacerbation of distress and symptom burden, with a higher risk of mortality (D'ippolito et al., 2017; Young et al., 2020). A recent study on 6,213 cancer patients showed that 23.4% of them reported depression, 17.7% anxiety, and 9.3% post-traumatic symptoms during the COVID-19 pandemic (Wang et al., 2020; Pigozzi et al., 2021). Moreover, another study analyzing the psychological burden of cancer patients before and after the pandemic highlighted higher levels of stress compared to non-cancer patients (Miaskowski et al., 2020). On the other hand, resilience has been described as a protective factor in life-threatening events and it plays a crucial role in determining how individuals react to and deal with stress. Resilience may be defined as the individual's ability to have biological, mental, and spiritual balance in the face of hazardous conditions, such as cancer and/or pandemics (Seiler and Jenewein, 2019). Moreover, patients often reported widespread concern and psychophysiological reactions related to stressful situations like the COVID-19 pandemic; they may also experience a psychophysiological hyperactivation, thus paying excessive attention to bodily sensations. Finally, hospitalization and management of acute complications of psychological problems increase the direct costs of treatments, while the increased disability may affect overall indirect costs due to the loss of productivity. The psychological burden can exacerbate the medical condition that in turn affects personal costs resulting from the loss of physical independence and the need for a carer, suddenly becoming a social cost (e.g. hospitalization, surgical and medical management, and institutionalization) (Sporinova et al., 2019).

Starting from these premises, there is an urgent need to address the enormous psychological burden of COVID-19 on those affected by cancer, while allowing the necessary social distancing. In this context, in-person participation was rapidly curtailed by the implementation of remote interventions able to facilitate stress management and enhance physical and emotional well-being (Trevino et al., 2021). Currently, in the field of eHealth treatments, the use of blended care is gaining visibility. The term “blended” refers to the combination of online and offline elements inside the same care-flow intervention. This “technology-supported” care combines unguided self-help modules with (scripted) face-to-face sessions. Offline and online components are interrelated methods that are strategically combined to build an intervention that merges the potential benefits of the two approaches (Wentzel et al., 2016). To date, the literature demonstrates that blended therapy displays encouraging effects (Wentzel et al., 2016) and can be used to deliver personal or automated feedback and support, synchronous or asynchronous guidance, and it can aid via online guidance or self-help units. The blended treatment could be innovatively provided through the implementation of online and offline relaxation techniques.

In this regard, previous studies demonstrated that interventions based on respiration and meditation helped individuals to alleviate the effects of stress by reducing physiologic arousal and restoring autonomic balance (Ditto et al., 2006; Wielgosz et al., 2019). Guided relaxation techniques can help people to achieve a deep state of relaxation through breathing exercises and focusing attention on body perception (e.g., body scan). The former may also be defined as “an efficient integrative body-mind training for dealing with stress, anxiety, and psychosomatic conditions”; it may help people slow their breathing, take in more oxygen, and reduce the use of shoulder, neck, and upper chest muscles, thus achieving better emotional balance and well-being (Porges, 2001). On the other hand, body scans aim to focus attention on different parts of the body and encourage awareness of the body's sensations, such as pain, tension, warmth, or relaxation. These techniques affect the physiological functions reducing hyperarousal and carry physical and psychological benefits: people learn how to actively modulate their breath and heartbeat, thus lowering anxiety and distress symptoms, and making room for positive thoughts and feelings (Zaccaro et al., 2018).

Forced social isolation will lead to the implementation of these practices using eHealth interventions. The usefulness of eHealth interventions is supported by studies showing that online relaxation interventions led to significant results as well as in-person interventions (Jung et al., 2016). Increasing the use of these therapies may lead to positive health outcomes and improve quality of life by preventing or delaying the onset of complications, reducing healthcare costs, and hospitalizations. The project's overall vision is to cost-effectively enhance relaxation interventions to reduce the effects of social isolation caused by the COVID-19 pandemic at both individual and societal levels and optimize health outcomes. The project will engage, train, and motivate patients by establishing the connection to raise awareness on the side effects of social isolation and improve their expertise on relaxation techniques. Achieving psychological and physical benefits, such as reducing heartbeat and stress activation or improving concentration, mood, and sleep quality are important factors that could be associated with the administration of pharmacological intervention and patient hospitalization. In fact, the use of non-pharmacological interventions, such as relaxation techniques, has been linked to greater clinical outcomes and a decrease in hospital admissions and healthcare costs (Ozamiz-Etxebarria et al., 2020). On the other hand, psychoeducational intervention showed significant results in cancer patients by improving disease care techniques and quality of life, reducing treatment-related discomfort, and lowering psychological burdens, such as distress, depression, and anxiety symptoms (Wu et al., 2018). Furthermore, the psychoeducational approach has been already applied as an active control group in internet-based protocols (Newby et al., 2018).

## Objective and Aims

The overall goal of the study is to provide an innovative intervention for cancer patients based on an on-line platform and help them to manage and prevent psychological problems related to social isolation. Specifically, the aim is to test and compare the efficacy of two web-based interventions (guided relaxation techniques vs. psychoeducational material) aimed at lowering stress in cancer patients.

## Materials and Methods

### Participants and Procedure

Participants will be enrolled through email. Those who will accept to take part in the study will be asked for the signature of the informed written consent. The study will receive ethical approval of the Ethical Committee of the University of the last author of the work. Then, participants will be randomly assigned to one of the two groups. In both groups, participants will receive on their email an invitation for an online platform where they will access the material. Participants will receive weekly reminders via e-mail.

A total of 150 participants (75 per group) will be enrolled based on the following inclusion criteria: (1) cancer patients (illness stage I–II); (2) aged from 18 to 65 years; (3) having an internet connection at home; (4) having a sufficient physical ability (auditory and visual ability) to use a remote internet device; (5) being a proficient Italian speaker; (6) providing the written informed consent. Patients (1) with neurological or psychiatric diseases, (2) hospitalized, and (3) with expertise in relaxation techniques or meditation will be excluded from the study.

Participation in the study will be voluntary, and participants will be allowed to withdraw from the study at any moment. The research protocol follows the CONSORT-EHEALTH V1.6 Guidelines (Eysenbach and CONSORT-EHEALTH Group, 2011). The study will be conducted according to the principles stated in the Declaration of Helsinki (59th WMA General Assembly, World Medical Association, 2008).

### Study Design and Measures

A between-subjects design will be adopted to administer one of two web-based interventions (guided relaxation techniques vs. psychoeducational material) to both cancer patients' groups. The web-based interventions will be composed of exercises on relaxation and meditation practices, presented in both automated content and interactive group sessions, or by fixed psychoeducational content.

In the blended intervention group, participants will be split into three sub-samples of 25 persons, and they will receive twice a week 1 audio of 10 min and a video clip of 15 min, with the guided exercise of relaxation or meditation. Plus, once a week, participants of the blended intervention group will be asked to participate in a 45 min group session with a psychotherapist that will guide some relaxation exercises. We decided to divide the sample into sub-samples of 25 participants to simplify the management of the groups. Indeed, conducting 45 min groups with more participants might lower the possibility of interaction between participants and with the psychotherapist. For example, the psychotherapist might ask for personal sharing of the experience or participants might have the necessity to ask questions.

In the psychoeducational group, participants will receive three times a week a link with psychoeducational content on stress, anxiety, hyperarousal, and social isolation (video, audio, and text). The materials used in the present study will be created *ad hoc* by the two first authors, in collaboration with a trained psychotherapist. A detailed description of the interventions is reported in Appendix A in Supplementary Material.

Along with the sociodemographic variables (age, gender, employment status, marital status, and level of education), all the participants will complete the following validated questionnaires:

- the Hospital Anxiety and Depression Scale (HADS) (Zigmond and Snaith, 1983) for assessing anxiety and depressive symptoms. It is a 14-items scale divided into two subscales ranging from 0 (not at all) to 3 (high presence of symptoms), with higher values indicating higher levels of anxiety and/or depression;
- the Distress Thermometer for measuring distress symptoms (Jacobsen et al., 2005; Donovan et al., 2014). It is a single-item tool with a Likert scale ranging from 0 (no distress) to 10 (extreme distress);
- the Impact of Event Scale-Revised version (IES-R) (Weiss, 2007) for assessing the reflect intrusion (eight items), avoidance (eight items), and hyperarousal (six items) related to traumatic events. It is a 22-item scale ranging from 0 (not at all) to 4 (extremely), with a higher score indicating higher distress in response to a specific traumatic event;
- the Connor-Davidson Resilience Scale (Connor and Davidson, 2003) for measuring the resilience level of cancer patients. It contains 25 items ranging from 1 to 5, with higher scores indicating higher resilience;
- the UCLA Loneliness Scale (Version 3) (Russell, 1996) for assessing social loneliness. It consists of 20-items ranging from 1 (Never) to 4 (Often), with a higher score indicating greater feelings of loneliness and social isolation.

All the previous questionnaires will be administered before the start of the interventions (T0). Two weeks (T1), 4 weeks (T2), and 2 months (T3) after the beginning of the interventions, participants will be asked to complete the same questionnaires.

### Sample Size Calculation and Statistical Analysis

We computed the required sample size with the G^*^Power 3.1.9.2 software (Faul et al., 2007; Erdfelder et al., 2009) for the *F*-test. The main endpoint will be the difference in the improvement of the overall score of the IES-R scale. A repeated measure ANOVA will be performed to test differences in the IES-R total scores between groups across the four-time points. The Type I error (α) rate was set at 0.05 (two-sided) and the Power (1 – β) was set at 0.95. The a priori correlation between repeated measures was set at 0.5 and the value of the F was set at 0.25 (*medium* effect size). We found a required sample of 132 subjects. To consider the possibility of attrition, we increased the sample by 14% of the required sample size. The total sample resulted in 150 participants (75 per group). Time will be considered a within-subjects variable, while the type of intervention will be considered a between-subject variable. *Post hoc* comparisons with the Tukey HSD-test will be performed and the effect sizes will be reported in the form of η^2^.

The same analyses will be repeated for all the questionnaires, except the one on the demographic variables. Indeed, as secondary analysis, differences in questionnaire scores between the two groups (i.e., HADS, Distress Thermometer, IES-R, Connor-Davidson Resilience Scale, the UCLA Loneliness Scale) will be assessed using the same analysis of the IES-R scale.

Group differences in questionnaires' measures will be analyzed with *T*-tests, while the chi-square statistic will be used to test the association between treatment groups and socio-demographic variables (gender, age, marital status, and employment status). Correlation analysis will be applied to assess the association between the questionnaires scores across all the time points.

Moreover, the scores of the HADS questionnaire will also be interpreted according to the clinical cutoffs (Annunziata et al., 2020). The scale score reliability (Cronbach α) will also be computed.

### Expected Results

We primarily hypothesize a greater improvement in the specific domain of stress symptoms (IES-R) assessed in the group receiving the interactive intervention, compared to the group which will receive only fully automated psychoeducational contents. Secondarily, we expect the same trend of improvement across all the psychological variables in the blended intervention group. Finally, we expect that the blended approach characterized by the presence of both interactive group and relaxation exercises will lead to a relief of the perceived social isolation.

## Discussion

Patients with cancer are immunocompromised and at higher risk of serious illness from infection. Therefore, people adopt behaviors aimed at protecting themselves, such as social distancing; on the other hand, feelings of loneliness and isolation may exacerbate and become burdensome. The limited exposure to social interactions with family, friends, and members of the community can have psychological consequences and lead to poorer mental well-being. A study of the general population also showed loneliness scores in the time of the COVID-19 pandemic resulted to be associated with depressive symptoms and suicidal ideation (Killgore et al., 2020). Furthermore, in cancer patients, delaying testing or clinical procedures might result in enhanced distress (Albano et al., 2020).

Implementing web-based practices on people who are forced into mandatory social isolation may help them become more aware of their mind-body condition and reduce negative effects (Pizzoli et al., 2020a,b). Moreover, relaxation techniques help individuals in achieving a greater state of well-being, increasing the ability to cope with stressful situations (resilience), and strengthening the immune system. Overcoming geographic and mobility problems, both patients and carers using telehealth tools reported a higher perception of social support and more empowerment (Kondylakis et al., 2017; Marzorati et al., 2018).

Remote psychotherapy, and specifically remote Cognitive-Behavioral Therapy, proved to be at least effective as the standard therapy while being less expensive and more accessible than the standard treatments (Richards et al., 2016; Thase et al., 2020). Furthermore, remote therapy might benefit from multiple technological sources to be implemented (e.g., telephone, video, email, audio).

Our results will also suffer from some limitations. Specifically, the comparison between the two groups leads to the analysis of differences between a fixed psychoeducational content and a more interactive and guided one. Thus we will be able to conclude only on the efficacy of a blended intervention vs. a fully automated one, not on the efficacy of the group sessions separately. Furthermore, we planned to include 31 participants per group in the sessions of the blended group, to render the groups more manageable. However, a smaller sample might be more effective and controllable.

There is also the chance that people with a higher propensity to use technology and the internet will agree to take part in the study. Thus, the enrolled sample might be more representative of those who already use technologies and have a good knowledge of digital tools. The same reason might be applied for those who have a positive attitude toward relaxation and meditation. Also, the participants included in the blended groups might be more engaged by groups sessions because of their attitude, thus limiting the generalizability of the results.

## Author Contributions

All authors listed have made a substantial, direct and intellectual contribution to the work, and approved it for publication.

## Conflict of Interest

The authors declare that the research was conducted in the absence of any commercial or financial relationships that could be construed as a potential conflict of interest.

## Publisher's Note

All claims expressed in this article are solely those of the authors and do not necessarily represent those of their affiliated organizations, or those of the publisher, the editors and the reviewers. Any product that may be evaluated in this article, or claim that may be made by its manufacturer, is not guaranteed or endorsed by the publisher.

## References

[B1] Albano D. Feraca M. Nemesure B. (2020). An assessment of distress levels of patients undergoing lung cancer treatment and surveillance during the COVID-19 pandemic. J. Nurse Pract. 17, 489–491. 10.1016/j.nurpra.2020.10.020 33250671 PMC7680042

[B2] Annunziata M. A. Muzzatti B. Bidoli E. Flaiban C. Bomben F. Piccinin M. . (2020). Hospital Anxiety and Depression Scale (HADS) accuracy in cancer patients. Support. Care Cancer 28, 3921–3926. 10.1007/s00520-019-05244-8 31858249

[B3] Connor K. M. Davidson J. R. T. (2003). Development of a new Resilience Scale: The Connor-Davidson Resilience scale (CD-RISC). Depress. Anxiety 18, 76–82. 10.1002/da.10113 12964174

[B4] D'ippolito S. Shams M. Ambrosini E. Cal I. G. Pastorelli D. (2017). The effect of loneliness on cancer mortality. Ann. Oncol. 28, vi82. 10.1093/annonc/mdx434

[B5] Ditto B. Eclache M. Goldman N. (2006). Short-term autonomic and cardiovascular effects of mindfulness body scan meditation. Ann. Behav. Med. 32, 227–234. 10.1207/s15324796abm3203_9 17107296

[B6] Donovan K. A. Grassi L. McGinty H. L. Jacobsen P. B. (2014). Validation of the distress thermometer worldwide: state of the science. Psychooncology 23, 241–250. 10.1002/pon.3430 25160838

[B7] Emami A. Javanmardi F. Pirbonyeh N. Akbari A. (2020). Prevalence of underlying diseases in hospitalized patients with COVID-19: a systematic review and meta-analysis. Arch. Acad. Emerg. Med. 8, e35. 10.22037/aaem.v8i1.600 32232218 PMC7096724

[B8] Erdfelder E. FAul F. Buchner A. Lang A. G. (2009). Statistical power analyses using G^*^Power 3.1: tests for correlation and regression analyses. Behav. Res. Methods 41, 1149–1160. 10.3758/BRM.41.4.1149 19897823

[B9] Eysenbach G. CONSORT-EHEALTH Group. (2011). CONSORT-EHEALTH: improving and standardizing evaluation reports of Web-based and mobile health interventions. J. Med. Internet Res. 13, e126. 10.2196/jmir.1923 22209829 PMC3278112

[B10] Faul F. Erdfelder E. Lang A. G. Buchner A. (2007). G^*^Power 3: a flexible statistical power analysis program for the social, behavioral, and biomedical sciences. Behav. Res. Methods 39, 175–191. 10.3758/BF03193146 17695343

[B11] Jacobsen P. B. Donovan K. A. Trask P. C. Fleishman S. B. Zabora J. Baker F. . (2005). Screening for psychologic distress in ambulatory cancer patients: a multicenter evaluation of the distress thermometer. Cancer 103, 1494–1502. 10.1002/cncr.20940 15726544

[B12] Jung Y.-H. Ha T. M. Oh C. Y. Lee U. S. Jang J. H. Kim J. . (2016). the effects of an online mind-body training program on stress, coping strategies, emotional intelligence, resilience and psychological state. PLoS ONE 11, e0159841. 10.1371/journal.pone.0159841 27479499 PMC4968838

[B13] Killgore W. D. S. Cloonan S. A. Taylor E. C. Dailey N. S. (2020). Loneliness: a signature mental health concern in the era of COVID-19. Psychiatry Res. 290, 113117. 10.1016/j.psychres.2020.113117 32480121 PMC7255345

[B14] Kondylakis H. Bucur A. Dong F. Renzi C. Manfrinati A. Graf N. . (2017). IManageCancer: developing a platform for empowering patients and strengthening self-management in cancer diseases, in Proceedings - IEEE Symposium on Computer-Based Medical Systems (Thessaloniki). 10.1109/CBMS.2017.62

[B15] Marzorati C. Renzi C. Russell-Edu S. W. Pravettoni G. (2018). Telemedicine use among caregivers of cancer patients: systematic review. J. Med. Internet Res. 20, e223. 10.2196/jmir.9812 29914858 PMC6028768

[B16] Miaskowski C. Paul S. M. Snowberg K. Abbott M. Borno H. Chang S. . (2020). Stress and symptom burden in oncology patients during the COVID-19 pandemic. J. Pain Symptom Manage. 60, e25–e34. 10.1016/j.jpainsymman.2020.08.037 32889039 PMC7462969

[B17] Newby J. M. Smith J. Uppal S. Mason E. Mahoney A. E. J. Andrews G. (2018). Internet-based cognitive behavioral therapy versus psychoeducation control for illness anxiety disorder and somatic symptom disorder: a randomized controlled trial. J. Consult. Clin. Psychol. 86, 89–98. 10.1037/ccp0000248 29172593

[B18] Ozamiz-Etxebarria N. Santa María M. D. Munitis A. E. Gorrotxategi M. P. (2020). Reduction of COVID-19 anxiety levels through relaxation techniques: a study carried out in northern spain on a sample of young University students. Front. Psychol. 11:2038. 10.3389/fpsyg.2020.02038 32982849 PMC7477108

[B19] Pigozzi E. Tregnago D. Costa L. Insolda J. Turati E. Rimondini M. . (2021). Psychological impact of COVID-19 pandemic on oncological patients: a survey in Northern Italy. PLoS ONE 16, e0248714. 10.1371/JOURNAL.PONE.0248714 33724999 PMC7963060

[B20] Pizzoli S. F. M. Marzorati C. Mazzoni D. Pravettoni G. (2020a). Web-based relaxation intervention for stress during social isolation: Randomized controlled trial. JMIR Ment. Heal. 7, e22757. 10.2196/22757 33200990 PMC8080491

[B21] Pizzoli S. M. F. Marzorati C. Mazzoni D. Pravettoni G. (2020b). An internet-based intervention to alleviate stress during social isolation with guided relaxation and meditation: protocol for a randomized controlled trial. JMIR Res. Protoc. 9:e19236. 10.2196/19236 32530814 PMC7301689

[B22] Porges S. W. (2001). The polyvagal theory: phylogenetic substrates of a social nervous system. in Int. J. Psychophysiol. 123–146. 10.1016/S0167-8760(01)00162-3 11587772

[B23] Richards D. A. Ekers D. McMillan D. Taylor R. S. Byford S. Warren F. C. . (2016). Cost and outcome of behavioural activation versus cognitive behavioural therapy for depression (COBRA): a randomised, controlled, non-inferiority trial. Lancet 388, 871–880. 10.1016/S0140-6736(16)31140-0 27461440 PMC5007415

[B24] Russell D. W. (1996). UCLA Loneliness Scale (Version 3): reliability, validity, and factor structure. J. Pers. Assess. 66, 20–40. 10.1207/s15327752jpa6601_2 8576833

[B25] Seiler A. Jenewein J. (2019). Resilience in cancer patients. Front. Psychiatry 10, 208. 10.3389/fpsyt.2019.00208 31024362 PMC6460045

[B26] Sigorski D. Sobczuk P. Osmola M. Kuć K. Walerzak A. Wilk M. . (2020). Impact of COVID-19 on anxiety levels among patients with cancer actively treated with systemic therapy. ESMO Open 5, e000970. 10.1136/esmoopen-2020-000970 33097653 PMC7590347

[B27] Sporinova B. Manns B. Tonelli M. Hemmelgarn B. Macmaster F. Mitchell N. . (2019). Association of mental health disorders with health care utilization and costs among adults with chronic disease. JAMA Netw. Open 2, e199910. 10.1001/jamanetworkopen.2019.9910 31441939 PMC6714022

[B28] Thase M. E. McCrone P. Barrett M. S. Eells T. D. Wisniewski S. R. Balasubramani G. K. . (2020). Improving cost-effectiveness and access to cognitive behavior therapy for depression: providing remote-ready, computer-assisted psychotherapy in times of crisis and beyond. Psychother. Psychosom. 89, 307–313. 10.1159/000508143 32396917 PMC7483890

[B29] Trevino K. M. Raghunathan N. Latte-Naor S. Polubriaginof F. C. G. Jensen C. Atkinson T. M. . (2021). Rapid deployment of virtual mind-body interventions during the COVID-19 outbreak: feasibility, acceptability, and implications for future care. Support. Care Cancer 29, 543–546. 10.1007/s00520-020-05740-2 32902712 PMC7479401

[B30] van de Haar J. Hoes L. R. Coles C. E. Seamon K. Fröhling S. Jäger D. . (2020). Caring for patients with cancer in the COVID-19 era. Nat. Med. 26, 665–671. 10.1038/s41591-020-0948-7 32405058

[B31] Wang Y. Duan Z. Ma Z. Mao Y. Li X. Wilson A. . (2020). Epidemiology of mental health problems among patients with cancer during COVID-19 pandemic. Transl. Psychiatry 10, 263. 10.1038/s41398-020-00950-y 32737292 PMC7393344

[B32] Weiss D. S. (2007). The impact of event scale: revised, in Cross-Cultural Assessment of Psychological Trauma and PTSD, eds J. P. Wilson and C. C. So-Kum Tang (Boston, MA: Springer US), 219–238. 10.1007/978-0-387-70990-1_10

[B33] Wentzel J. van der Vaart R. Bohlmeijer E. T. van Gemert-Pijnen J. E. W. C. (2016). Mixing online and face-to-face therapy: how to benefit from blended care in mental health care. JMIR Ment. Heal. 3, e9. 10.2196/mental.4534 26860537 PMC4764785

[B34] Wielgosz J. Goldberg S. B. Kral T. R. A. Dunne J. D. Davidson R. J. (2019). Mindfulness meditation and psychopathology. Annu. Rev. Clin. Psychol. 15, 285–316. 10.1146/annurev-clinpsy-021815-093423 30525995 PMC6597263

[B35] World Medical Association (2008). Declaration of Helsinki. Ethical principles for medical research involving human subjects. Seoul: 59th WMA General Assembly.

[B36] Wu P. H. Chen S. W. Huang W. T. Chang S. C. Hsu M. C. (2018). Effects of a psychoeducational intervention in patients with breast cancer undergoing chemotherapy. J. Nurs. Res. 26, 266–279. 10.1097/jnr.0000000000000252 29360672

[B37] Young A. M. Ashbury F. D. Schapira L. Scotté F. Ripamonti C. I. Olver I. N. (2020). Uncertainty upon uncertainty: supportive care for cancer and COVID-19. Support. Care Cancer 28, 4001–4004. 10.1007/s00520-020-05604-9 32613372 PMC7329359

[B38] Zaccaro A. Piarulli A. Laurino M. Garbella E. Menicucci D. Neri B. . (2018). How breath-control can change your life: a systematic review on psycho-physiological correlates of slow breathing. Front. Hum. Neurosci. 12, 353. 10.3389/fnhum.2018.00353 30245619 PMC6137615

[B39] Zigmond A. S. Snaith R. P. (1983). The hospital anxiety and depression scale. Acta Psychiatr. Scand. 67, 361–370. 10.1111/j.1600-0447.1983.tb09716.x 6880820

