# Supplementary material for: Remote Relaxation and Acceptance Training for the Management of Stress in Cancer Patients: A Study Protocol
Source: Front Psychol. 2021 Oct 13;12:710861. doi: 10.3389/fpsyg.2021.710861 (PMC8548685; doi:10.3389/fpsyg.2021.710861)
Supplement: Supplementary file 1 [file Data_Sheet_1.docx]

**Appendix A**

| *Timeline* | *Topic* | *Intervention* | |
| --- | --- | --- | --- |
|  |  | **Blended intervention group** | **Psychoeducational group** |
| T0 |  | Questionnaires | Questionnaires |
|  |  |  |  |
| Week 1 | Stress | -Video Clip 1 (15 min): *“Awareness of stress and awareness of breathing”*  -Audio Clip 2 (10 min): *“Square breathing guided exercise”*  -Group of patients | -Video Clip 1 (15 min): “*What is stress*”  -Audio Clip 2 (10 min): “*How to cope with stress*”  - Written Text on Stress with a 3 questions quiz |
|  |  |  |  |
| T1  Week 2 | Anxiety | -Video Clip 1 (15 min): *“Feel anxiety and breathing exercise”*  -Audio Clip 2 (10 min): *“Meditation on thoughts and anxiety”*  -Group of patients | -Video Clip 1 (15 min): “*What is anxiety*”  -Audio Clip 2 (10 min): “*How to face anxiety*”  - Written Text on Anxiety with a 3 questions quiz |
|  |  |  |  |
| Week 3 | Arousal and hyper-arousal | -Video Clip 1 (15 min): *“Imagining the Safe Place”*  -Audio Clip 2 (10 min): *“Grounding exercise”*  Group of patients | -Video Clip 1 (15 min): “*What is arousal and when it becomes hyperarousal*”  -Audio Clip 2 (10 min): “*How to cope with hyperarousal*”  - Written Text on Hyperarousal with a 3 questions quiz |
|  |  |  |  |
| T2  Week 4 | Social Isolation | -Video Clip 1 (15 min): *“Guided meditation on the thoughts and emotions related to loneliness”*  -Audio Clip 2 (10 min): *“Positive guided imagery on solitude”*  Group of patients | -Video Clip 1 (15 min): *“What is social isolation and which feelings are usually associate with it”*  -Audio Clip 2 (10 min): “*Strategies to cope with social isolation and loneliness*”  - Written text on Social Isolation with a 3 questions quiz |
|  |  |  |  |
| T3  Week 8 |  | Questionnaires | Questionnaires |
